# Occurrence and Antimicrobial Resistance among Staphylococci Isolated from the Skin Microbiota of Healthy Goats and Sheep

**DOI:** 10.3390/antibiotics12111594

**Published:** 2023-11-05

**Authors:** Maria Wesołowska, Ewa Szczuka

**Affiliations:** Department of Microbiology, Institute of Experimental Biology, Faculty of Biology, Adam Mickiewicz University in Poznań, Uniwersytetu Poznańskiego 6, 61-614 Poznań, Poland; maria.wesolowska@amu.edu.pl

**Keywords:** staphylococci, skin microbiota of sheep and goats, antimicrobial resistance, One Health

## Abstract

Staphylococci colonize the skin and mucous membranes of different animals. The purpose of this study was to determine the staphylococcal composition of the skin microbiota of healthy, non-vet visiting, and antimicrobially non-treated sheep and goats. In total, 83 strains (44 from goats and 39 from sheep) were isolated and identified using matrix-assisted laser desorption ionization time of flight mass spectrometry (MALDI-TOF MS). The diversity of the isolated *Staphylococcus* species was relatively high, and only coagulase-negative staphylococci (CoNS) were isolated. In sheep, *S. vitulinus* (9/39, 23.1%) was the most common species, followed by *S. equorum* (8/39, 20.5%), *S. lentus* (7/39, 17.9%), *S. sciuri* (6/39, 15.4%), *S. xylosus* (6/39, 15.4%), *S. warneri* (1/39, 2.6%), *S. simulans* (1/39, 2.6%), and *S. nepalensis* (1/39, 2.6%). In the goats, the most common species was *S. sciuri*, which was detected in 13 (29.5%) animals. The goat skin was also inhabited by *S. equorum* (7/44, 15.9%), *S. vitulinus* (6/44, 13.6%), *S. cohnii* (5/44, 11.4%), *S. lentus* (4/44, 9.1%), *S. suscinus* (3/44, 6.8%), *S. caprae*, (2/44, 4.5%), *S. auricularis* (2/44, 4.5%), *S. warneri* (1/44, 2.3%), and *S. xylosus* (1/44, 2.3%). Only one *S. xylosus* strain of goat origin carried the enterotoxin gene (*sea*). Antimicrobial resistance was not common among the isolated staphylococci. Only 31 (37.3%) strains were resistant to at least one antimicrobial agent, with the highest frequency of resistance to penicillin (16.8%), followed by clindamycin (9.6%), erythromycin (8.4%), moxifloxacin (8.4%), and tetracycline (7.2%). All isolates were susceptible to eight antibiotics (amikacin, gentamycin, ciprofloxacin, levofloxacin, rifampicin, chloramphenicol, trimethoprim-sulfamethoxazole, and tigecycline), representing six different classes. Three isolates displayed a multi-resistance phenotype (MDR): the goat isolates *S. cohnii* and *S. sciuri*, as well as the ewe isolate *S. xylosus*. The MDR *S. cohnii* isolate was found to be methicillin-resistant and carried the *mecA* gene. Moreover, the staphylococci isolated from the healthy animals carried genes conferring resistance to β-lactams (*mecA*, *blaZ*), tetracyclines (*tetL*, *tetK*), macrolides (*ermB*, *ermC*), lincosamides (*lnu*), and fluoroquinolones (*grlA*). However, the prevalence of these genes was low.

## 1. Introduction

Staphylococci are commonly found on the skin of humans, other mammals, and birds. There are differences in the composition of staphylococci in the skin microbiota between animals and humans. The coagulase-negative staphylococci (CoNS) *Staphylococcus epidermidis* is the most abundant species on human skin. The coagulase-positive staphylococci (CoPS) *S. intermedius* and *S. pseudintermedius* are the most prevalent bacteria in the skin microbiota of domestic animals (dogs and cats), whereas *S. lentus* is the most common species in healthy pigeons [1,2,3]. Little is known about staphylococci isolated from the skin microbiota of healthy ewes and goats. Previous studies have focused on *Staphylococcus strains* associated with skin infections [4]. Folliculitis, furunculosis, and impetigo are common staphylococcal infections in goats and sheep. Of note, folliculitis and furunculosis infections may be manifested by skin lesions in any location on the body [5]. Staphylococcal infections usually occur upon bacterial entrance through the skin and breakdown of mucous membranes, e.g., during injuries or surgical incisions. The invading bacteria proliferate locally and produce enzymes and toxins that cause tissue damage [6]. In sheep and goats, the most common cause of staphylococcal skin disease is *S. aureus*, although other bacteria, e.g., *S. hyicus*, *S. haemolyticus*, *S. warneri*, *S. epidermidis*, *S. chromogenes*, *S. caprae*, *S. simulans*, and *S. xylosus*, have also been isolated from some cases of disease [7]. Furthermore, *S. aureus* and other coagulase-positive staphylococcal species: *S. hyicus*, *S. intermedius*, and *S. schleiferi* are associated with clinical mastitis in ewes [8]. Also, coagulase-negative staphylococci, *S. epidermidis*, *S. saprophyticus*, *S. simulans*, *S. xylosus*, *S. warneri*, and *S. chromogenes*, have been isolated from cases of clinical mastitis [6] (Supplementary Materials Table S1). These bacteria can contaminate raw food and produce staphylococcal enterotoxins (SEs) that can cause food poisoning. Most staphylococcal food-borne diseases are caused by the production of classical enterotoxins (SEA, SEB, SEC, SED, and SEE). Generally, staphylococcal food poisoning (SFP) is a self-limited illness resolving within 24–48 h after the onset. However, the SFP symptoms in some groups may require hospitalization, especially in the case of infants, the elderly, or immunocompromised individuals [9].

Recent studies have reported increased resistance to β-lactams, tetracyclines, clindamycin, and less frequently, fluoroquinolones. The major threat to human and animal health associated with staphylococci is their methicillin resistance. Methicillin resistance associated with carriage of the *mecA* or *mecC* genes confers resistance to β-lactam antimicrobials. These genes are located on a mobile genetic element, i.e., the staphylococcal cassette chromosome *mec* (SCC*mec*), enabling horizontal transmission between isolates. Notably, methicillin-resistant staphylococci (MRS) are often multi-drug-resistant (MDR; resistant to three or more classes of non-beta lactam antibiotics), which extremely limits therapeutic options [10,11]. Recent studies have shown cross-transmission of methicillin-resistant staphylococci between goats and humans [12]. This aspect cannot be ignored, as food-producing animals play an important role in the transmission of MDR bacteria through the food chain. Moreover, microbiome transfer may occur through direct contact between animals and humans. Therefore, livestock animals are a crucial part of One Health [13].

This study was focused on commensal staphylococci in the skin microbiota of healthy sheep and goats. Moreover, another aim was to determine whether the animal skin was colonized by methicillin-resistant *Staphylococcus* sp. (MRS).

## 2. Results

The research included 44 staphylococcal strains from the skin of goats and 39 from sheep. Staphylococci were detected on the skin of each animal. The diversity of the *Staphylococcus* species was relatively high. In total, 18 different species, all belonging to the CNS, were identified. In sheep, *S. vitulinus* (9/39, 23.1%) was the most common species, followed by *S. equorum* (8/39, 20.5%), *S. lentus* (7/39, 17.9%), *S. sciuri* (6/39, 15,3%), *S. xylosus* (6/39, 15.4%), *S. warneri* (1/39, 2.6%), *S. simulans* (1/39, 2.6%), and *S. nepalensis* (1/39, 2.6%). In the goats, the most common was *S. sciuri*, which was detected in 13 (29.5%) animals. The goat skin was also inhabited by *S. equorum* (7/44, 15.9%), *S. vitulinus* (6/44, 13.6%), *S. cohnii* (5/44, 11.4%), *S. lentus* (4/44, 9.1%), *S. suscinus* (3/44, 6.8%), *S. caprae*, (2/44, 4.5%), *S. auricularis* (2/44, 4.5%), *S. warneri* (1/44, 2.3%), and *S. xylosus* (1/44, 2.3%) (Table S2).

Overall, 37.3% of the isolated strains were resistant to at least one antibiotic. The highest resistance rate was detected for penicillin (16.8%), clindamycin (9.6%), erythromycin (8.4%), moxifloxacin (8.4%), and tetracycline (7.2%) (Figure S1). The detailed results of antimicrobial resistance are presented in Table 1 and Table 2. Of note, 100% of the strains exhibited susceptibility to gentamycin, amikacin, rifampicin, ciprofloxacin, levofloxacin, chloramphenicol, trimethoprim/sulfamethoxazole, and tigecycline. All of the penicillin-resistant strains in the disk diffusion method were positive for the presence of the *blaZ* gene. Only one strain (*S. cohnii* isolated from the goats) presented a methicillin resistance phenotype. The strain was resistant to cefoxitin in the disk diffusion method and the harbored *mecA* gene. Three strains were multidrug resistant (MDR): *S. cohnii* was resistant to cefoxitin, penicillin, tetracycline, and tobramycin; *S. sciuri* was resistant to moxifloxacin, erythromycin, and clindamycin; and *S. xylosus* was resistant to penicillin, erythromycin, and clindamycin. The profiles of antimicrobial resistance and resistance genes detected in each strain are presented in Table A1 and Table A2. Interestingly, resistance-coding genes were also detected in strains isolated from the goats presenting intermediate susceptibility to erythromycin (1 *S. equorum* strain with the *ermB* gene detected) and clindamycin (2 *S. sciuri* strains with the *lnu* gene detected).

All the *Staphylococcus* strains tested negative for enterotoxin-coding genes except for one *S. xylosus* strain isolated from sheep where the *sea* gene was detected.

## 3. Discussion

This is the first study incorporating MALDI-TOF-MS to successfully characterize commensal staphylococcal populations in healthy sheep and goats. It should be emphasized that these were no-vet visiting and antimicrobially non-treated animals. We isolated 83 staphylococci and were able to assign all the isolates to 18 different species. The diversity of the *Staphylococcus* species isolated from the skin microbiota of these animals was relatively high. Ten different staphylococcal species (i.e., *S. sciuri*, *S. equorum*, *S. vitulinus*, *S. cohnii*, *S. lentus*, *S. caprae*, *S. suscinus*, *S. auricularis*, *S. warneri*, and *S. xylosus)* were detected in the goats, while eight species (i.e., *S. vitulinus*, *S. equorum*, *S. lentus*, *S sciuri*, *S. xylosus*, *S. warneri*, *S. simulans*, and *S. nepalensis*) were identified in the sheep. A previous study reported only two species, i.e., *S. aureus* and *S. epidermidis*, isolated from the body sides of healthy sheep and goats [14]. In another study, *S. lentus*, *S. aureus*, *S. epidermidis*, *S. xylosus*, and *S. caprae* were isolated from the nasal cavity, vagina, udder, and anus of dairy goats. However, these animals suffered from mastitis. The most prevalent species was *S. lentus* [15]. In this study, *S. sciuri* was the dominant bacterial skin colonizer of goats, whereas *S. vitulinus* was the most frequently isolated from the ewe skin microbiota. Of note, the phylogenomic analyses of the *Staphylococcaceae* family suggest the taxonomic reassignment of five *Staphylococcus* species, i.e., *S*. *sciuri*, *S*. *fleurettii*, *S*. *lentus*, *S*. *stepanovicii*, and *S*. *vitulinus*, to the novel genus *Mammaliicoccus*, with *Mammaliicoccus sciuri* as the type species [16]. As mentioned above, *S. sciuri*, *S. vitulinus*, and *S*. *lentus* (proposed to be reclassified as *Mammaliicoccus*) were common species of the animal skin microbiota. Additionally, *S. equorum* were isolated frequently from the ewe and goat skin. We observed differences in the staphylococcal composition between sheep and goat. *S. cohnii*, *S. suscinus*, *S. caprae* and *S. auricularis* were present only on the goat skin. In contrast, *S. simulans* and *S. nepalensis* were found only in the ewe’s skin. Noteworthy, the healthy skin was not colonized by CPS. It is well known that *S. aureus* and other CPS, particularly *S. schleiferi and S. pseudintermedius*, as well as coagulase-variable *S. hyicus*, are important veterinary pathogens responsible for infections in a number of different animal species [7]. Nasal carriage of *S. aureus* was documented among sheep and goats treated at the University of Veterinary Medicine in Austria [17]. Also, a study from France showed that 29% of ewes carried *S. aureus* in their nares [18].

Another focus of this study was the identification of enterotoxin genes (*sea*) in staphylococci colonizing non-hospitalized animals. We found the enterotoxin gene only in one *S xylosus* strain, isolated from the goats. In a previous study, no enterotoxin genes were detected in any CNS strains isolated from goats with mastitis; however, *sec* and *see* were identified in *S. aureus* [15]. In another study, the *see* gene was found in a single *S. lentus* strain isolated from goats’ milk [19]. More recently, Kotzamanidis et al. [20] reported the presence of enterotoxin genes in *S. aureus* strains recovered from clinical mastitis and subclinical mastitis cases in goats, sheep, and bovines.

In this study, the majority of commensal CoNS isolates from the skin microbiota of healthy animals were susceptible to a broad range of antimicrobials. All the isolates were susceptible to eight antibiotics (amikacin, gentamycin, ciprofloxacin, levofloxacin, rifampicin, chloramphenicol, trimethoprim-sulfamethoxazole, and tigecycline), representing six different classes. Overall, we found that 41% of the isolates were susceptible to all the tested antimicrobials, and only 3.6% of the isolates exhibited multi-resistance. Among all the isolates, 26 (37.34%) were found to be resistant to at least one antimicrobial agent, with the highest frequency of resistance to penicillin, followed by clindamycin, erythromycin, moxifloxacin, and tetracycline. A single *S. cohnii* isolate was methicillin-resistant. Of note, the ewe isolates exhibited the highest frequency of resistance to penicillin and tetracycline compared with the goat isolates. A higher frequency of resistance to penicillin and tetracycline was observed in a previous study in Greece [20], but these investigations focused on *S. aureus* strains isolated from mastitis. A study from Taiwan also reported a higher rate of resistance to penicillin among staphylococci strains isolated from cases of mastitis in dairy goats [15]. These strains showed the high frequency of resistance to tetracycline, neomycin, and gentamycin. A study from Tunisia indicated that the majority of *S. aureus* strains isolated from healthy sheep were susceptible to tested antimicrobial agents, with the following exceptions: penicillin, tetracycline, and fusidic acid [21].

Screening for genes encoding resistance to many classes of antimicrobial agents, such as penicillin, cephalosporins, tetracyclines, macrolides, lincosamides, and aminoglycosides, indicated their presence in only 11 staphylococcal strains. Among sheep isolates, *tek(K)* gene encoding membrane-associated efflux proteins conferring resistance to tetracycline was detected in two *S. lentus* strains. The *tetL* gene was present in a single *S. cohnii* isolate from the goats. Moreover, the *erm* (*B*) and *erm* (*C*) genes, which code for methylases that modify the target site in 23S rRNA and inhibit the binding of macrolides, lincosamides, and streptogramin B MLS_B_ to the bacterial ribosome, were present in the goat *S. equorum* and *S. sciuri* isolates. The presence of the *ermC* gene in a *S. sciuri* isolate from sheep has previously been reported [22]. In another study, the resistance gene *mphC*-coding macrolide phosphotransferase was found in a *S. lentus* isolate of ewers origin [23]. In this study, the gene *lnu(A)* encoding a lincosamide nucleotidyltransferase was detected in two *S. lentus* isolates from goats. Only one *S. vitulinus* isolate carried *grlA*, which codes for resistance to phenicols. The *blaZ* gene encoded β-lactamases were identified in three strains of goat’s origin, i.e., *S. cohnii*, *S. sciuri*, and *S. warneri* and in ten strains of ewe’s origin, i.e., *S. equorum*, *S. lentus*, *S. sciuri*, *S. vitulinus*, *and S. xylosus.* Gharsa et al. [21] reported the presence of *blaZ* as well as *tet(K)* genes in *Staphylococcus aureus* isolated from nasal swabs of healthy sheep. Importantly, methicillin resistance conferred by the gene *mec A* was found in one *S. cohnii* isolate colonizing the goats. A study focused on *S. aureus* reported the presence of the *mecA* gene in five strains isolated from healthy sheep [23]. Another study from Austria reported the presence of the *mecA* gene in 12 strains isolated from hospitalized goats [17]. More recently, El-Deep et al. [12] identified seven methicillin-resistant *S. aureus* and two methicillin-resistant *S. epidermidis* strains.

To summarize, resistance to tetracyclines and penicillin was found among the staphylococci isolated from healthy sheep and goats. It is important because these antibiotics are used as first-line treatments in veterinary medicine. However, no resistance to trimethoprim-sulfamethoxazole, which is commonly used in veterinary practice, was detected. Also, all staphylococci strains were susceptible to gentamycin, amikacin, and ciprofloxacin, i.e., antibiotics that are clinically effective in veterinary medicine. Moreover, the majority of stains were susceptible to erythromycin, which is used to treat staphylococcal infections in animals.

In the One Health concept, the health of humans is closely connected to the health of animals. In addition to direct contact between animals and humans, antimicrobial-resistant bacteria and resistance genes may be transmitted via the food chain. The probability of transferring staphylococci to milk is high. Moreover, farm animals can be a source of microorganisms moving into their environment, which may lead to significant changes in the farm dust microbiome. The present study indicated that non-vet visiting goats and sheep from small farms are colonized by antibiotic-resistant staphylococci, including multidrug-resistant strains. However, the prevalence of antibiotic resistance genes among these strains was low. This study showed that healthy sheep and goats are colonized by a variety of CoNS staphylococci.

## 4. Material and Methods

### 4.1. Bacterial Isolation and Identification 

Samples were collected from animals bred on small farms by private owners. The animals were not exposed to antimicrobial treatment prior to this study. Swabs were taken from the skin in the perianal area using Amies transport medium swabs (Sarstedt, Hildesheim, Germany). One swab was collected from each animal. The samples were inoculated on Chapman Agar (BIomeriux, Marcy-l’Étoile, France). After 24 h of incubation at 37 °C, bacterial colonies with proper morphological features were isolated on Columbia Agar (BIomeriux, Marcy-l’Étoile, France ) and then cultivated according to standard microbiological procedures. Staphylococcal strains were identified to the species level using MALDI TOF-MS (Matrix-assisted laser desorption/ionization–time of flight mass spectrometry) following the manufacturer’s procedure in the research mode (MALDI Biotyper^®^, Bruker, Billerica, MA, USA).

### 4.2. Susceptibility Testing

Susceptibility to all antimicrobial agents was tested using the disk-diffusion method according to the Clinical and Laboratory Standards Institute (CLSI) guidelines, with the exception of amikacin, tobramycin, and tigecycline, which followed EUCAST guidelines. The following antimicrobial agents were tested: cefoxitin, ciprofloxacin, levofloxacin, moxifloxacin, clindamycin, chloramphenicol, erythromycin, gentamicin, penicillin, rifampicin, tetracycline, trimethoprim/sulfamethoxazole, amikacin, tobramycin, and tigecycline.

### 4.3. Preparation of Total DNA for PCR and Detection of SE Genes and Antibiotic Resistance Genes

DNA from *Staphylococcus* strains was isolated and purified using the Genomic Mini DNA kit (A&A Biotechnology, Gdynia, Poland). The presence of se genes (*sea*, *seb*, *sec*, *sed*, *see*) and antibiotic resistance genes *blaZ*, *mecA*, *tetK*, *tetM*, *tetL*, *tetO*, *aac(6՛)/aph(2՛՛)*, *aph(30)-IIIa*, *ant(40)-Ia*, *erm(A)*, *erm(B)*, *erm(C)*, *msr(A)*, *lun(A)*, *gyrA*, *gyrB*, and *grlA* was assessed using PCR assays as described previously [24,25,26,27,28,29].

## Figures and Tables

**Table 1:** Antimicrobial resistance of staphylococcal strains isolated from the skin of goats.

Antibiotic Class	Antimicrobial Drug	Antimicrobial Susceptibility					
		Resistant		Intermediate		Sensitive	
		n	%	n	%	n	%
β-lactams	Cefoxitin	1	2.27	0	0.00	43	97.72
	Penicillin	4	9.09	0	0.00	40	90.09
Macrolides	Erythromycin	4	9.09	6	13.95	34	77.27
Lincosamides	Clindamycin	4	9.09	8	18.18	32	72.72
Tetracycline	Tetracycline	2	4.54	0	0.00	42	95.45
Aminoglycosides	amikacin	0	0.00	0	0.00	44	100.00
	gentamycin	0	0.00	0	0.00	44	100.00
	tobramycin	1	2.27	0	0.00	43	97.72
Fluoroquinolones	Ciprofloxacin	0	0.00	0	0.00	44	100.00
	Levofloxacin	0	0.00	0	0.00	44	100.00
	Moxifloxacin	7	15.90	4	9.30	33	75.00
Ryfamicins	Rifampicin	0	0.00	0	0.00	44	100.00
Chloramphenicol	Chloramphenicol	0	0.00	0	0.00	44	100.00
Sulphonamides	Trimetoprim-sulfametoksaole	0	0.00	0	0.00	44	100.00
Glycylcyclines	Tigecycline	0	0.00	0	0.00	44	100.00

**Table 2:** Antimicrobial resistance of staphylococcal strains isolated from the skin of sheep.

Antibiotic Class	Antimicrobial Drug	Antimicrobial Susceptibility					
		Resistant		Intermediate		Sensitive	
		n	%	n	%	n	%
β-lactams	Cefoxitin	0	0.00	0	0.00	39	100.00
	Penicillin	9	23.07	0	0.00	30	76.92
Macrolides	Erythromycin	3	7.69	5	12.82	31	79.48
Lincosamides	Clindamycin	4	10.25	6	15.38	29	74.35
Tetracycline	Tetracycline	4	10.25	0	0.00	35	89.74
Aminoglycosides	amikacin	0	0.00	0	0.00	39	100.00
	gentamycin	0	0.00	0	0.00	39	100.00
	tobramycin	0	0.00	0	0.00	39	100.00
Fluoroquinolones	Ciprofloxacin	0	0.00	0	0.00	39	100.00
	Levofloxacin	0	0.00	0	0.00	39	100.00
	Moxifloxacin	0	0.00	1	2.56	38	97.43
Ryfamicins	Rifampicin	0	0.00	0	0.00	39	100.00
Chloramphenicol	Chloramphenicol	0	0.00	0	0.00	39	100.00
Sulphonamides	Trimetoprim-sulfametoksaole	0	0.00	0	0.00	39	100.00
Glycylcyclines	Tigecycline	0	0.00	0	0.00	39	100.00

## Data Availability

Not applicable.

## Supplementary Materials

The following supporting information can be downloaded at: https://www.mdpi.com/article/10.3390/antibiotics12111594/s1, Table S1: The staphylococcal species isolated from the skin of sheep and goats as described in previous reports; Table S2: *Stahylococcus* species isolated from goats and sheep. Figure S1: Resistance to antibiotics in *Staphylococcus* strains isolated from sheep and goats.

## Appendix

**Table A1:** Antimicrobial resistance profiles and distribution of resistance genes in staphylococcal isolates of goat origin.

Strain	Isolate ID	Antimicrobial Resistance Profile	Number Indicating the Prevalence of Antibiotic Resistance Genes (No *)																
			*mecA*	*blaZ*	*tetK*	*tetM*	*tetL*	*tetO*	*aac(6՛)/* *aph(2՛՛)*	*aph(30)-IIIa*	*ant(40)-Ia*	*ermA*	*ermB*	*ermC*	*msr(A)*	*lnu(A)*	*gyrA*	*gyrB*	*grl(A)*
*S. caprae*	G04	MOX, P	0	1	0	0	0	0	0	0	0	0	0	0	0	0	0	0	0
*S. conhnii*	G09	Fox, P, Tet, Tob	1	0	0	0	1	0	0	0	0	0	0	0	0	0	0	0	0
*S. equorum*	G10	Tet	0	0	0	0	0	0	0	0	0	0	0	0	0	0	0	0	0
*S. equorum*	G14	ERY	0	0	0	0	0	0	0	0	0	0	0	0	0	0	0	0	0
*S. lentus*	G17	DA	0	0	0	0	0	0	0	0	0	0	0	0	0	0	0	0	0
*S. lentus*	G19	ERY, DA	0	0	0	0	0	0	0	0	0	0	0	0	0	0	0	0	0
*S. lentus*	G20	DA	0	0	0	0	0	0	0	0	0	0	0	0	0	0	0	0	0
*S. sciuri*	G27	MOX	0	0	0	0	0	0	0	0	0	0	0	0	0	0	0	0	0
*S. sciuri*	G29	P, Mox	0	1	0	0	0	0	0	0	0	0	0	0	0	0	0	0	0
*S. sciuri*	G31	ERY, DA, MOX	0	0	0	0	0	0	0	0	0	0	0	1	0	0	0	0	0
*S. vitulinus*	G40	MOX	0	0	0	0	0	0	0	0	0	0	0	0	0	0	0	0	0
*S. vitulinus*	G41	MOX	0	0	0	0	0	0	0	0	0	0	0	0	0	0	0	0	0
*S. vitulinus*	G42	MOX	0	0	0	0	0	0	0	0	0	0	0	0	0	0	0	0	1
*S. warneri*	G43	P, ERY	0	1	0	0	0	0	0	0	0	0	0	0	0	0	0	0	0

* Number indicating the prevalence of antibiotic resistance genes in staphylococci strains. P, penicillin; ERY, erythromycin; Tet, tetracycline; DA, clindamycin; MOX, moxifloxacin. Tob, tobramycin; Fox, cefoxitin.

**Table A2:** Antimicrobial resistance profiles and distribution of resistance genes in isolated staphylococcal isolates of ewe origin.

Strain	Isolate ID	Antimicro- bial Resistance Profile	Number Indicating the Prevalence of Antibiotic Resistance Genes (No *)																
			*mecA*	*blaZ*	*tetK*	*tetM*	*tetL*	*tetO*	*aac(6՛)/* *aph(2՛՛)*	*aph(30)-IIIa*	*ant(40)-Ia*	*ermA*	*ermB*	*ermC*	*msr(A)*	*lnu(A)*	*gyrA*	*gyrB*	*grl(A)*
*S. equorum*	S01	P	0	1	0	0	0	0	0	0	0	0	0	0	0	0	0	0	0
*S. equorum*	S07	P	0	1	0	0	0	0	0	0	0	0	0	0	0	0	0	0	0
*S. equorum*	S08	ERY	0	0	0	0	0	0	0	0	0	0	0	0	0	0	0	0	0
*S. lentus*	S09	P, Tet	0	1	0	0	0	0	0	0	0	0	0	0	0	0	0	0	0
*S. lentus*	S10	DA	0	0	0	0	0	0	0	0	0	0	0	0	0	0	0	0	0
*S. lentus*	S11	Tet	0	0	0	0	0	0	0	0	0	0	0	0	0	0	0	0	0
*S. lentus*	S12	DA	0	0	0	0	0	0	0	0	0	0	0	0	0	0	0	0	0
*S. lentus*	S14	Tet	0	0	1	0	0	0	0	0	0	0	0	0	0	0	0	0	0
*S. nepalensis*	S16	Tet	0	0	1	0	0	0	0	0	0	0	0	0	0	0	0	0	0
*S. sciuri*	S17	P	0	1	0	0	0	0	0	0	0	0	0	0	0	0	0	0	0
*S. scuri*	S18	P	0	1	0	0	0	0	0	0	0	0	0	0	0	0	0	0	0
*S. sciuri*	S21	ERY	0	0	0	0	0	0	0	0	0	0	0	0	0	0	0	0	0
*S. vitulinus*	S32	P	0	1	0	0	0	0	0	0	0	0	0	0	0	0	0	0	0
*S. xylosus*	S34	P	0	1	0	0	0	0	0	0	0	0	0	0	0	0	0	0	0
*S. xylosus*	S35	ERY, DA, P	0	1	0	0	0	0	0	0	0	0	0	0	0	0	0	0	0
*S. xylosus*	S36	DA, P	0	1	0	0	0	0	0	0	0	0	0	0	0	0	0	0	0
*S. xylosus*	S39	P	0	1	0	0	0	0	0	0	0	0	0	0	0	0	0	0	0

* Number indicating the prevalence of antibiotic resistance genes in staphylococci strains. P, penicillin; ERY, erythromycin; Tet, tetracycline; DA, clindamycin.

## References

[1] Byrd A.L., Belkaid Y., Segre J.A. (2018). The human skin microbiome. Nat. Rev. Microbiol..

[2] Ross A.A., Hoffmann A.R., Neufeld J.D. (2019). The skin microbiome of vertebrates. Microbiome.

[3] Szczuka E., Wesołowska M., Krawiec A., Kosicki J.Z. (2023). Staphylococcal species composition in the skin microbiota of domestic pigeons (Columba livia domestica). PLoS ONE.

[4] Hofman A.R. (2017). The cutaneous ecosystem: The roles of the skin microbiome in health and its association with inflammatory skin conditions in humans and animals. Vet. Dermatol..

[5] Mala L., Lalouckova K., Skrivanova E. (2021). Bacterial skin infections in livestock and plant-based alternatives to their antibiotic treatment. Animals.

[6] Schmidt T., Kock M.M., Ehlers M.M., Ossiprandi M.C. (2015). Antimicrobial Resistance in staphylococci at the human—Animal interface. Antimicrobial Resistance.

[7] Foster A.P. (2012). Staphylococcal skin disease in livestock. Vet. Dermatol..

[8] Vasileiou N.G.C., Chatzopoulos D.C., Sarrou S., Fragkou I.A., Katsafadou A.I., Mavrogianni V.S., Petinaki E., Fthenakis G.C. (2019). Role of staphylococci in mastitis in sheep. J. Dairy Res..

[9] Fisher E.L., Otto M., Cheung G.Y.C. (2018). Basis of virulence in enterotoxin-mediated staphylococcal food poisoning. Front. Microbiol..

[10] Schwarz S., Feßler A.F., Loncaric I., Wu C., Kadlec K., Wang Y., Shen J. (2018). Antimicrobial resistance among staphylococci of animal origin. J. Microbiol. Spectr..

[11] Argudín M.A., Deplano A., Meghraoui A., Dodémont M., Heinrichs A., Denis O., Nonhoff C., Roisin S. (2017). Bacteria from animals as a pool of antimicrobial resistance genes. Antibiotics.

[12] El-Deeb W., Cave R., Fayez M., Alhumam N., Quadri S., Mkrtchyan H.V. (2022). Methicillin resistant staphylococci isolated from goats and their farm environments in Saudi Arabia genotypically linked to known human clinical isolates: A pilot study. Microbiol. Spectr..

[13] Trinh P., Zaneveld J.R., Safranek S., Rabinowitz P.M. (2018). One Health Relationships Between Human, Animal, and Environmental Microbiomes: A Mini-Review. Front. Public Health.

[14] Kayalvizhi N., Anthony T., Gunasekaran P. (2008). Characterization of the predominant bacteria associated with sheep and goat skin. JALCA.

[15] Chu C., Yu C., Lee Y., Su Y. (2012). Genetically divergent methicillin-resistant *Staphylococcus aureus* and *sec*-dependent mastitis of dairy goats in Taiwan. BMC Vet. Res..

[16] Madhaiyan M., Wirth J.S., Saravanan V.S. (2020). Phylogenomic analyses of the *Staphylococcaceae* family suggest the reclassification of five species within the genus *Staphylococcus* as heterotypic synonyms, the promotion of five subspecies to novel species, the taxonomic reassignment of five *Staphylococcus* species to *Mammaliicoccus* gen. nov., and the formal assignment of *Nosocomiicoccus* to the family *Staphylococcaceae*. Int. J. Syst. Evol. Microbiol..

[17] Schauer B., Krametter-Frötscher R., Knauer F., Ehricht R., Monecke S., Feßler A.T., Schwarz S., Grunert T., Spergser J., Loncaric I. (2018). Diversity of methicillin-resistant *Staphylococcus aureus* (MRSA) isolated from Austrian ruminants and New World camelids. Vet. Microbiol..

[18] Vautor E., Abadie G., Guibert J.-M., Chevalier N., Pépin M. (2005). Nasal carriage of *Staphylococcus aureus* in dairy sheep. Vet. Microbiol..

[19] Dakić I., Vuković D., Stepanović S., Hauschild T., Ježek P., Petráš P., Morrison D. (2005). Survey of genes encoding staphylococcal enterotoxins, toxic shock syndrome Toxin 1, and exfoliative toxins in members of the *Staphylococcus sciuri* Group. J. Clin. Microbiol..

[20] Kotzamanidis C., Vafeas G., Giantzi V., Anastasiadou S., Mygdalias S., Malousi A., Loukia E., Daniel S., Zdragas A. (2021). *Staphylococcus aureus* Isolated from ruminants with mastitis in Northern Greece dairy Herds: Genetic relatedness and phenotypic and genotypic characterization. Toxins.

[21] Gharsa H., Ben Slama K., Lozano C., Gómez-Sanz E., Klibi N., Ben Sallem R., Gómez P., Zarazaga M., Boudabous A., Torres C. (2012). Prevalence, antibiotic resistance, virulence traits and genetic lineages of *Staphylococcus aureus* in healthy sheep in Tunisia. Vet. Microbiol..

[22] Lodder G., Werckenthin C., Schwarz S., Dyke K. (1997). Molecular analysis of naturally occuring *ermC*-encoding plasmids in staphylococci isolated from animals with and without previous contact with macrolide/lincosamide antibiotics. FEMS Immunol. Med. Microbiol..

[23] Schwendener S., Perreten V. (2012). New MLSB resistance gene erm (43) in *Staphylococcus lentus*. Antimicrob. Agents Chemother..

[24] Sawant A.A., Gillespie B.E., Oliver S.P. (2009). Antimicrobial susceptibility of coagulase-negative *Staphylococcus* species isolated from bovine milk. Vet. Microbiol..

[25] Nawaz M., SKhan S.A., Khan A.A., Khambaty F.M., Cerniglia C.E. (2000). Comparative molecular analysis of erythromycin-resistance determinants in staphylococcal isolates of poultry and human origin. Mol. Cell. Probes.

[26] Chajęcka-Wierzchowska W., Zadernowska A., Nalepa B., Sierpińska M., Łaniewska-Trokenheim L. (2015). Coagulase-negative staphylococci (CoNS) isolated from ready-to-eat food of animal origin--phenotypic and genotypic antibiotic resistance. Food Microbiol..

[27] Gómez-Sanz E., Torres C., Lozano C., Fernández-Pérez R., Aspiroz C., Ruiz-Larrea F., Zarazaga M. (2010). Detection, molecular characterization, and clonal diversity of methicillin-resistant *Staphylococcus aureus* CC398 and CC97 in Spanish slaughter pigs of different age groups. Foodborne Pathog. Dis..

[28] Ardic N., Sareyyupoglu B., Ozyurt M., Haznedaroglu T., Ilga U. (2006). Investigation of aminoglycoside modifying enzyme genes in methicillin-resistant staphylococci. Microbiol. Res..

[29] Osman K.M., Amer A.M., Badr J.M., Helmy N.M., Elhelw R.A., Orabi A., Bakry M., Saad A.S.A. (2016). Antimicrobial resistance, biofilm formation and *mecA* characterization of methicillin-susceptible *S. aureus* and Non-*S. aureus* of beef meat origin in Egypt. Front. Microbiol..

