# Supplementary material for: Occurrence and Antimicrobial Resistance among Staphylococci Isolated from the Skin Microbiota of Healthy Goats and Sheep
Source: Antibiotics (Basel). 2023 Nov 5;12(11):1594. doi: 10.3390/antibiotics12111594 (PMC10668681; doi:10.3390/antibiotics12111594)
Supplement: Supplementary file 1 [file antibiotics-12-01594-s001.zip › antibiotics-2682921-supplementary.pdf]

Supplementary Materials

Table S1. The staphylococcal species isolated from the skin of sheep and goats as described in previous reports.

Staphylococcal species	Animal host species	Disorders/Healthy skin	References
S. aureus	Sheep, Goat	Skin infections	[7]
	Sheep	Mastitis	[8,20]
	Goat	Mastitis	[20,15]
	Sheep, Goat	Healthy skin	[14]
S. hyicus	Sheep, Goat	Skin infections	[7]
	Sheep	Mastitis	[8]
S. haemolyticus	Sheep, Goat	Skin infections	[7]
S. warneri	Sheep, Goat	Skin infections	[7]
	Sheep	Mastitis	[8]
S. epidermidis	Sheep, Goat	Skin infections	[7]
	Sheep	Mastitis	[8]
		Healthy skin	[14]
S. chromogenes	Sheep, Goat	Skin infections	[7]
	Sheep,	Mastitis	[8]
S. caprae	Sheep, Goat	Skin infections	[7]
S. simulans	Sheep, Goat	Skin infections	[7]
	Sheep	Mastitis	[8]
S. xylosus	Sheep, Goat	Skin infections	[7]
	Sheep	Mastitis	[8]
S. intermedius	Sheep	Mastitis	[8]
S. schleiferi	Sheep	Mastitis	[8]
S. saprophyticus	Sheep	Mastitis	[8]
S. lentus	Sheep, Goat	Mastitis	[7,22]
S. cohnii	Goat	Mastitis	[7]
S. equorum	Goat	Mastitis	[7]

Table S2. *Stahylococcus* species isolated from goats and sheep.

***Stapylococcus* species**	**Number of identified strains**			
	**Goats**		**Sheep**	
	n	%	n	%
S. auricularis	2	4.54	0	0.00
S. caprae	2	4.54	0	0.00
S. conhnii	5	11.36	0	0.00
S. equorum	7	15.90	8	20.51
S. lentus	4	9.09	7	17.94
S. nepalensis	0	0.00	1	2.56
S. sciuri	13	29.54	6	15.38
S. simulans	0	0.00	1	2.56
S. succinus	3	6.81	0	0.00
S. vitulinus	6	13.36	9	23.07
S. warneri	1	2.27	1	2.56
S. xylosus	1	2.27	6	15.38

n -number of strains.

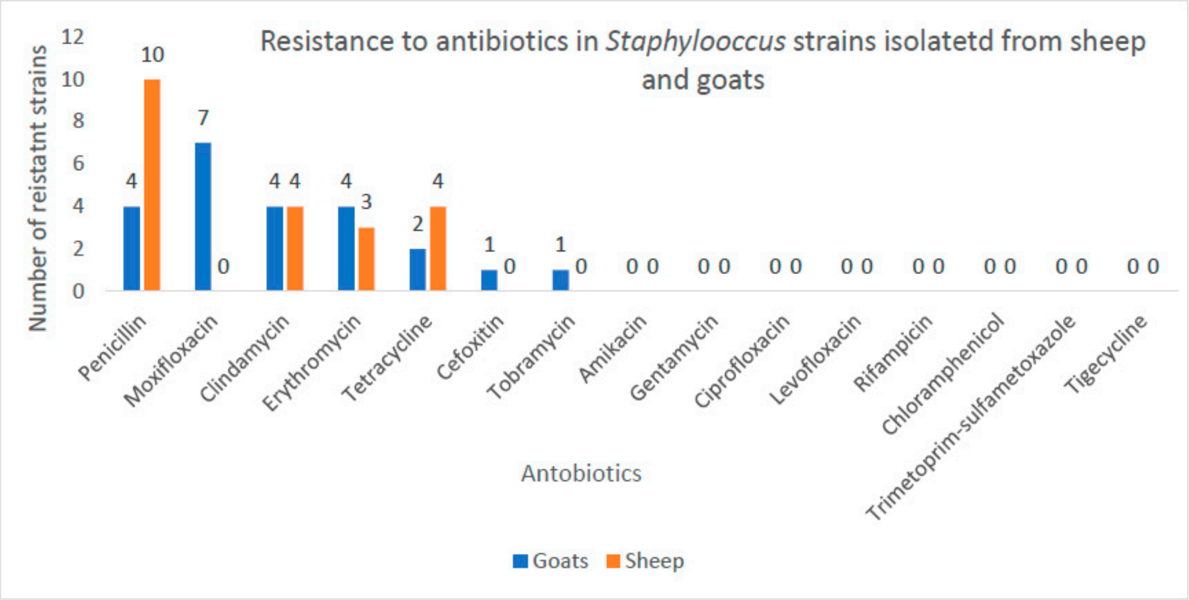

Figure S1. Resistance to antibiotics in *Staphylococcus* strains isolated from sheep and goats.
